# Endometriosis III and IV as a risk factor for tubal obstruction in infertile women

**DOI:** 10.5935/1518-0557.20190004

**Published:** 2019

**Authors:** Fabiana C Approbato, Mario S Approbato, Diego F Rezende, Tatiana M Silva, Yanna A R Lima, Beatriz Bacheschi do Carmo Benetti

**Affiliations:** 1 Reproduction Human Laboratory. Obstetrics and Gynecology Dept. Federal University of Goiás State, Brazil; 2 Nutrition Academic, Federal University of Goias State, Brazil

**Keywords:** endometriosis, tubal obstruction, tubal occlusion, infertility

## Abstract

**Objective:**

A previous study carried out among infertile women with tubal obstruction identified a relative risk of 2.5 for *Chlamydia trachomatis* seropositivity. However, endometriosis may also be associated with increased risk. This study aimed to evaluate the risk of tubal obstruction associated with endometriosis III/IV among women submitted to assisted reproductive procedures.

**Methods:**

A case-control study was performed among 144 women with and without tubal obstruction. We calculated the odds ratio with 95% CI regarding the association of endometriosis III/IV and tubal obstruction. Calculations were performed using the SPSS v.17.0 package.

**Results:**

The mean age was 33.7 years (4.76 SD), and the mean infertility duration time was 66.7 months (120.6 SD). The total prevalence of endometriosis was 20/144 (13%). Among 144 women, the risk group with tubal obstruction and endometriosis III/IV comprised 7out of 20 (35%), compared with the group without such risk, that comprised 22 out of 124 (17%). The X^2^ test was 3.19 with a *p*-value of 0.07. The odds ratio (OR) was 2.5 (95% CI: 0.647-9.639).

**Conclusion:**

Although the OR was 2.5, there was no significant difference between the groups with and without endometriosis III/IV. Further studies are needed to increase the sample size.

## INTRODUCTION

The prevalence of infertility in patients with endometriosis varies depending on the author. Some have reported that 25-50% of women with infertility have endometriosis, and about 30-50% of the women with endometriosis have infertility (Hickey *et al.*, 2014). Between 10 and 15 % of all women seek IVF in the UK due to tubal infertility (HFEA, 2016). In this cases, the cause may be pelvic inflammatory disease, endometriosis, salpingitis isthmica nodosa, polyps and surgical trauma (Honoré *et al.,* 1999). Tanahatoe *et al.* (2003) found 10 % of infertility in endometriosis. These authors also found that the most important causes of infertility in couples are ovulation disorders, tubal obstruction and semen abnormalities (mainly azoospermia, oligozoospermia, teratozoospermia and astenozoospermia). This causes account for approximately 75% of infertility in couples. The remaining is so far unknown (Tanahatoe *et al.,* 2003). Mild or moderate endometriosis is related to subfertility with pregnancy rates of 17.7% at nine months of follow-up. Endometriosis is a net factor of subfertility, mainly in stages III and IV. In a series of cases, a fertility rate of 3% has been reported after 12 months in cases of stage IV endometriosis (Marcoux *et al.,* 1997).

It is a disease that can affect several organs, such as the pelvic peritoneum, fallopian tubes, ovaries, subcutaneous tissue, umbilicus, urinary tract, bladder, heart, kidney, lung, liver, pancreas, muscles, central nervous system, among others, which makes it a multi-systemic disease (Goldberg & Bedaiwy, 2007; Lee *et al.,* 2008). Endometriotic lesions are more frequent in the peritoneum and pelvic organs, especially in the ovaries, followed by the recto-vaginal septum. It is found less frequently in extra-pelvic regions, such as gastrointestinal (sigmoid, rectum, ileocecal and appendix) and urinary tract, extremities, subcutaneous tissue and abdominal wall (Lee *et al.,* 2008). The mechanism of impaired fertility in endometriosis may involve anatomical distortions in the pelvis, adhesions, endometriomas or the production of substances (prostaglandins, cytokines, and growth factors) that are harmful to normal ovarian function, ovulation, fertilization and implantation. The really valid mechanisms are tubal obstruction, pelvic adhesions and ovarian endometriomas that distort anatomical relationships, limit the access of oocytes and spermatozoa and alter fimbriae mobility, mainly in stages III and IV (Mahutte & Arici, 2002). Phenomena such as anovulation, endocrine dysfunction, luteinized unruptured follicle syndrome, inadequate luteal phase, autoimmune dysfunction, abnormalities of the ovule quality and sperm alterations are theoretical mechanisms, still unproven, used to explain infertility in endometriosis in stages I and II (Toya *et al.,* 2000). However, the two most probable mechanisms to explain the infertility in these stages are maturing on the late follicular phase and the antispermatic effect impairing folliculogenesis with oocyte alterations.

There are very few publications about the effect of endometriosis on tubal permeability. Some time ago Bowman & Cooke (1994) found that there was a strong correlation between the degree of intratubal damage and the extent of pelvic adhesions when the etiology was a previous pelvic inflammatory disease (PID), but not when the underlying etiology was endometriosis. However, in the endometriosis subgroup, intraluminal ampullary pathology was noted in 3 of 11 tubes (27%) assessed, and intraluminal fimbrial pathology was noted in 4 of 11 tubes (36%) assessed.

Osuga *et al.* (2008) describe a case of a patient with endometriosis who sought infertility treatment. During ovarian stimulation, an image of hydrosalpinx without infection appears and changed dramatically in size with the menstrual cycle. The patient was 32 years old and had had endometriosis since 24 years of age. She underwent ethanol sclerotherapy of a bilateral ovarian endometrioma at age 26 and laparoscopic cystectomy for ovarian endometrioma at age 30. Serum *Chlamydia trachomatis* IgA and IgM antibodies were negative. During ultrasonography work-up to check follicular growth and ovulation, the author noticed a hydrosalpinx-like structure that appeared larger at each ultrasound scan. This structure was minimal during the menstrual period. It would reach its maximum size during ovulation, and then shrank again. A later laparoscopy revealed endometriosis and tubal obstruction. Salpingectomy was undertaken to improve the IVF-ET outcome. Histologically, they found endometriosis at the tubal wall serosa layer.

## MATERIAL AND METHODS

A case-control study was performed, involving 144 women with and without tubal obstruction. We calculated the odds ratio, with a 95% CI, of the patients with endometriosis III/IV having tubal obstruction. Calculations were performed using the SPSS package v.17.0. The statistical test was the Chi Square, with a *p* value of 0.05.

## RESULTS

**Figure 1:**
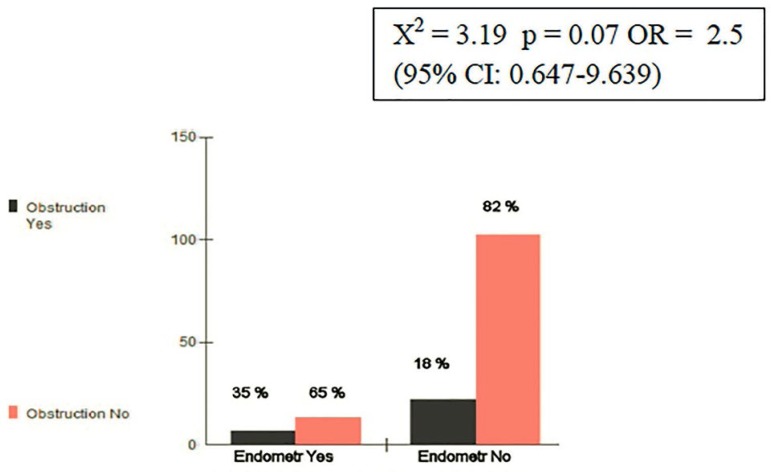
Endometriosis and Tubal Obstruction

The mean age of the patients was 33.7 years (4.76 SD). The mean infertility duration time was 66.7 months (120.6 SD). The endometriosis prevalence was 20/144 (13%). Among 144 women, the risk group (endometriosis II/IV) with tubal obstruction comprised 7out of 20 (35%), compared with the group without risk that comprised 22 out of 124 (17%). The X^2^ test was 3.19 with a *p*-value of 0.07. The odds ratio (OR) was 2.5 (95% CI: 0.647-9.639) (Figure 1).

## DISCUSSION

Due to diagnostic difficulties and the different types, the literature has few high quality publications on endometriosis. Broeze *et al.* (2012) for example, state that this disease is a condition that may result in tubal pathology, but information on endometriosis was either not documented in the original databases or not reported in a standardized way, or was in sufficient detail. For these reasons the author could even included endometriosis as a clinical variable in a meta-analysis.

Removing endometriomas without hydrosalpinx or tubal obstruction remains controversial. Some authors state that this procedure did not improve the results of *in vitro* fertilization (Garcia-Velasco *et al.,* 2004). Nevertheless assisted reproductive technology is better than surgery, and should be offered as a first-line treatment (Feinberg *et al.,* 2008).

Osuga *et al.* (2008) published a case of hydrosalpinx and endometrioma without apparent infection. Salpingectomy was undertaken to improve the IVF-ET outcome. However most of the hydrosalpinx was an infection sequel, mainly Chlamydia. This publication did not find an association between endometriosis III and IV and tubal obstruction, thought the statistical test almost reached significance. Further studies with larger data sets are needed to check these results.

## CONCLUSION

Although the OR was 2.5 (*p*=0.07) there was no significant difference between the groups with and without endometriosis III/IV. Further studies with larger samples are needed.
